# Arabic‐speaking male immigrants' perceptions of preventive initiatives: An interview study

**DOI:** 10.1111/hex.13766

**Published:** 2023-04-24

**Authors:** Marie Dahl, Susanne F. Søndergaard, Rafel Salman Al‐Allaq, Axel Diederichsen, Jes S. Lindholt

**Affiliations:** ^1^ Vascular Research Unit, Department of Vascular Surgery Viborg Regional Hospital Viborg Denmark; ^2^ Department of Clinical Medicine Aarhus University Aarhus Denmark; ^3^ Research Unit of Cardiac, Thoracic, and Vascular surgery, Department of Clinical Research Faculty of Health Sciences University of Southern Denmark Odense Denmark; ^4^ Centre for Research in Clinical Nursing, Viborg Regional Hospital and School of Nursing, VIA University Collage Viborg Denmark; ^5^ Department of Public Health, Nursing Aarhus University Aarhus Denmark; ^6^ Department of Science in Public Health University of Southern Denmark Odense Denmark; ^7^ Department of Cardiology Odense University Hospital Odense Denmark; ^8^ Department of Cardiothoracic and Vascular Surgery Odense University Hospital Odense Denmark; ^9^ Elitary Research Centre of Individualized Medicine in Arterial Disease (CIMA) Odense Denmark; ^10^ Cardiovascular Centre of Excellence in Southern Denmark (CAVAC) Odense Denmark

**Keywords:** cardiovascular disease, healthcare disparities, immigrants, men, prevention, public involvement, screening

## Abstract

**Background:**

Arabic‐speaking men are a sparsely investigated population in health promotion and disease prevention. This may hamper their ability to achieve the highest obtainable health due to less accessibility and acceptability of preventive measures.

**Aim:**

We explored Arabic‐speaking (Palestinian, Iraqi and Somali) male immigrants' perceptions of preventive initiatives in general and such initiatives for cardiovascular diseases (CVD) in particular to understand how to address inequalities in engagement in prevention.

**Methods:**

This qualitative study employed content analysis of semistructured interviews with 60–66‐year‐old Arabic‐speaking men living in Denmark. Supplementary, structured data, for example, health data, were collected. From June to August 2020, 10 men were interviewed.

**Findings:**

Preventive initiatives were found ethically and culturally acceptable alongside personally and socially relevant; they were perceived as humanitarian and caring for the participants' health, respecting of their self‐determination and enabling their empowerment. Thus, the participants entreated that their fellow countrymen be assisted in achieving the prerequisite coping capabilities to address inequality in access, perceived acceptance and relevance. This led us to define one main category ‘Preventive initiatives ‐ Caring and humanitarian aid empower us’ with the underlying subcategories: ‘We are both hampered and strengthened by our basic assumptions’ and ‘We need help to achieve coping capabilities enabling us to engage in preventive initiatives’.

**Conclusion:**

Prevention was perceived as acceptable and relevant. Even so, Arabic‐speaking men may be a hard‐to‐reach group due to their basic assumptions and impaired capabilities for engaging in prevention. Addressing inequality in accessibility, acceptability and relevance in regard to prevention may be promoted through a person‐centred approach embracing invitees' preferences, needs and values; and by strengthening invitees' health literacy through efforts at the structural, health professional and individual levels.

**Public Contribution:**

This study was based on interviews. The interviewees were recruited as public representatives to assist us in building an understanding of Arabic‐speaking male immigrants' perceptions of preventive initiatives in general and preventive initiatives for CVD in particular.

## 1 INTRODUCTION

According to the World Health Organization (WHO), cardiovascular diseases (CVD) account for the majority of deaths from noncommunicable diseases,^1^ and efforts are therefore warranted to prevent CVD. Preventive initiatives include ‘measures to reduce the occurrence of risk factors, prevent the occurrence of disease, to arrest its progress and reduce its consequences once established’.^2^ Preventive initiatives should also embrace health promotion strategies empowering people, especially those most marginalized.^3^ In prevention, empowerment is a process whereby people achieve greater control over decisions and actions influencing their health.^2^

Preventive initiatives must ensure equal opportunities for the target population and allow them to enjoy their highest attainable level of health.^4^ Thus, preventive initiatives must be acceptable and accessible. Acceptability refers to being ethically and culturally appropriate and being person‐centred while catering to target group needs, preferences and values.^3^, ^4^ In screening programmes, acceptability refers to, for example, the acceptance rate and whether the target population finds the proposed prevention efforts relevant.^5^ Accessibility implies that preventive initiatives are accessible to all without discrimination.^4^ However, nonparticipation in preventive initiatives is more prevalent amongst people with low income, low levels of education and amongst immigrants.^6^, ^7^, ^8^

In Denmark, disease prevention is publicly financed and equal access to health services is a healthcare cornerstone. Disease prevention rests mainly on opportunistic risk assessment generally performed by general practitioners (GPs) although systematic screening for cancer is offered. Recently, further efforts to prevent CVD have been in focus in research evaluating the effect of systematic cardiovascular screening.^9^, ^10^, ^11^ The Danish trial ‘Fighting Social Inequality in Cardiovascular Health’ (FISICH) randomised men in their 60s to screening for various CVDs to reduce social inequality in cardiovascular health.^12^ After the trial, FISICH was supplemented with the present qualitative immigrant study.

Qualitative research exploring acceptability showed that Danish women declined cardiovascular screening as they found it personally irrelevant^13^; a perception embedded in personal beliefs like feeling healthy, low perceived risk and relying on being capable of feeling whether something is brewing or not.^13^ Amongst Danish women, previous unfavourable experiences with the healthcare system may also influence screening acceptance.^13^ Similarly, Abdelmessih and colleagues found that Arabic‐speaking immigrants with poor trust in healthcare systems expressed reluctance towards seeking medical care despite having a CVD diagnosis.^14^ Moreover, they found that taking into account language proficiency and health literacy was essential for accessibility.^14^ However, these issues are sparsely investigated from ethnic minorities' perspectives, in particular within the context of early CVD detection. This is concerning as such knowledge is central for inclusive policies addressing ethnicity‐based inequality in access and health. Thus, to bridge this knowledge gap, we explored Arabic‐speaking (Palestinian, Iraqi and Somali) male immigrants' perception of preventive initiatives in general while focusing on cardiovascular screening, in particular, to understand how to address inequalities in engagement in prevention.

## 2 METHODS

We conducted a qualitative study with semi‐structured individual telephone interviews supplemented by structured data; telephone interviews are an acknowledged approach to exploring perceptions of a sensitive nature without personal confrontation.^15^ We followed the consolidated criteria for reporting qualitative research (Supporting Information Material: S1).^16^

### 2.1 Participants and recruitment

Eligible interview participants were purposively selected after the trial amongst men with Arabic names in a control arm of the cardiovascular screening programme, FISICH. In brief, FISICH randomised men in their 60s to screening for various CVDs.^12^ In the present study, potential participants were invited by surface mail. Accepting and cancelling were possible by text message, phone or e‐mail. All received information about FISICH as a proposed prevention strategy and were informed about the present study's aim. Initially, 12 responded, but after the interviews, 2 withdrew their consent as they were concerned that their signature might be abused by the authorities.

### 2.2 Data collection

A study‐specific semistructured interview guide was developed; informed by ethical aspects related to preventive services^5^, ^17^ and literature on ethnic minorities' perceptions of preventive initiatives (Supporting Information Material: S2). Three pilot interviews were conducted amongst Arabic‐speaking men to test the interview guide and strengthen the interviewer's interviewing skills. The pilot interviews were excluded from the study. Structured data were collected on, for example, cardiovascular morbidity, use of medicine and smoking. Notes were taken after each interview on, for example, important unrecorded statements and interview language. Each participant was interviewed once by R. S. A.‐A. supervised by M. D.

Data were collected from June to August 2020. The interviews, including obtaining verbal consent, lasted 19–53 min. All interviews were held in Arabic, tape‐recorded and transcribed verbatim by the interviewer. We followed the method of medical translation^18^ to verify the translation from Arabic into English. Transcripts were verified by a bilingual health professional.

### 2.3 Data analysis

**Figure 1:**
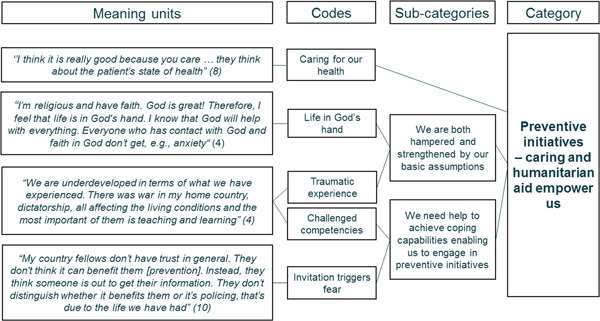
Illustration of the analysis process.

We conducted an interpretive, inductive content analysis; a recognized approach for achieving replicable and valid inferences from contextual data and producing new understandings followed by practical guides to action.^19^ The first author and the interviewer read the transcribed and translated interviews while collecting the data. As the last 2 interviews provided no new perspectives to the analysis, we deemed data saturation had been reached.^19^ Next, the transcribed interviews were reread to build an overall impression of the data. We (M. D. and R. S. A.‐A.) then identified units of analysis and conducted dialogue meetings to verify the meaning. Subsequently, the first author synthesized coded contents into subcategories. This abstraction process was iterative, shifting between raw data, coded contents and subcategories: it also included dialogue meetings with an expert in qualitative research (S. F. S.) until a consensus was reached on data interpretations and sufficient data abstraction. Finally, the main category and underlying subcategories were discussed with the research team. The interviewees provided no feedback on transcripts or findings. The analysis was facilitated by using NVivo 12 (International Pty). Figure 1 illustrates the analysis process with an example of the coding tree.

As 2 participants withdrew their written consent due to confidentiality preferences, the interviews were reanalyzed; even with the withdrawn interviews, we verified data saturation.

### 2.4 Research group

The research group comprised experts within the field of nursing and medicine with and without involvement in cardiovascular screening. This ensured the representation of various perspectives and the study's trustworthiness. The interviewer was a female bilingual and bicultural health science student of Arabic origin. No one knew the interview participants beforehand.

## 3 FINDINGS

Ten Arabic‐speaking 60–66‐year‐old male immigrants participated; all had lived in Denmark for 20–36 years and held residence permits. Table 1 provides selected information from the structured data; information that contributed to the in‐depth analysis.

**Table 1:** Participant characteristics.

Participant	Cardiovascular risk factors	Completed level of education	Country of origin	Danish language skills
1	Smoking, diabetes	Secondary	Iraq	No Danish skills
2	Smoking, diabetes	Secondary	Iraq	Danish skills
3	Previous smoker, heart surgery, hypertension, diabetes	Secondary	Palestine	No Danish skills
4	Heart surgery, hypertension	Unknown	Iraq	No Danish skills
5	None	Vocational	Iraq	Danish skills
6	High cholesterol	Secondary	Somalia	Danish skills
7	Smoking hookah	Vocational	Palestine	Danish skills
8	Smoking, hypertension	Tertiary	Palestine	Limited Danish skills
9	None	Tertiary	Iraq	Danish skills
10	None	Tertiary	Palestine	Danish skills

**Figure 2:**
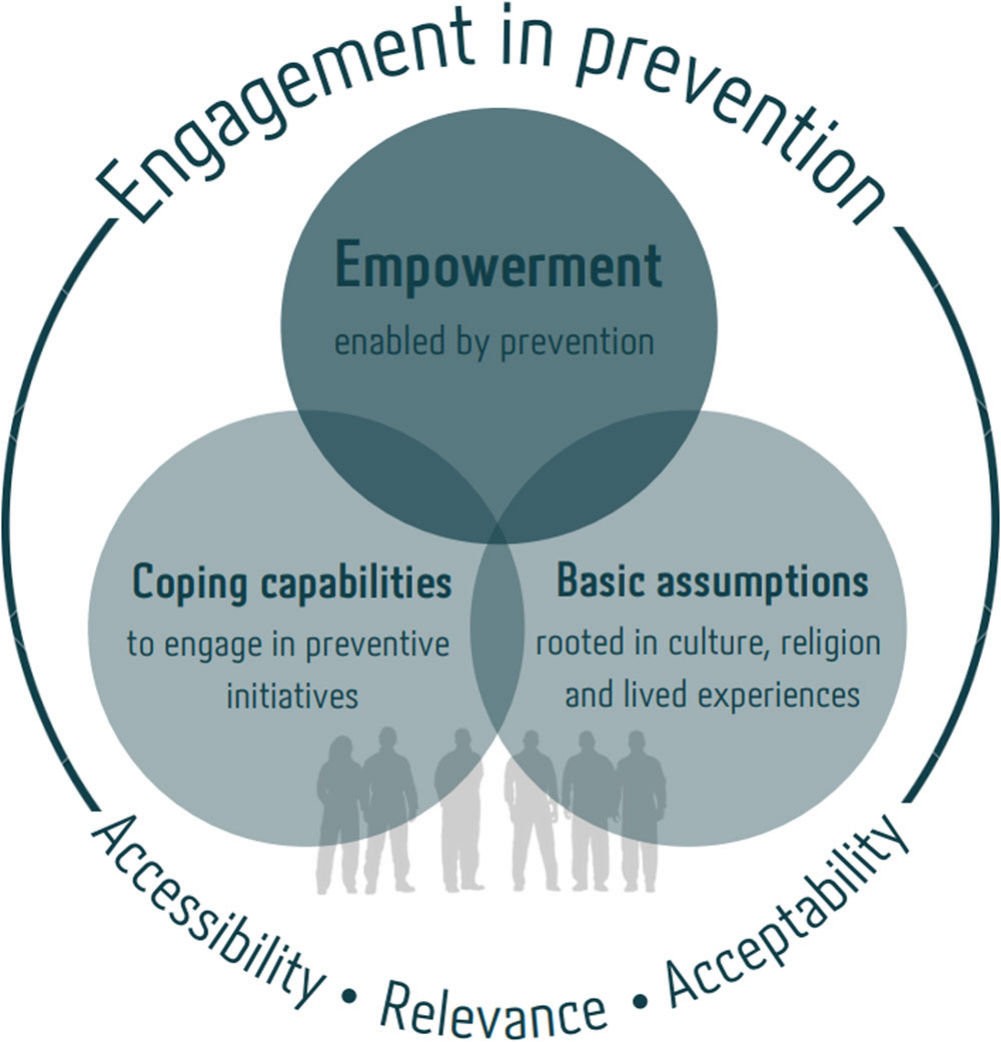
Factors influencing engagement in preventive initiatives in terms of accessibility, acceptability and relevance.

In our analysis, we identified one main category ‘Preventive initiatives ‐ Caring and humanitarian aid empower us’ with the underlying subcategories: ‘We are both hampered and strengthened by our basic assumptions’ and ‘We need help to achieve coping capabilities enabling us to engage in preventive initiatives’. Figure 2 shows that inviting Arabic‐speaking men to preventive initiatives demands awareness of how to improve their feeling of empowerment by strengthening their coping capabilities and acknowledging their basic assumptions. Empowerment, basic assumptions and coping capabilities were interrelated factors for engagement in prevention.

### 3.1 Preventive initiatives—Caring and humanitarian aid empower us

Preventive initiatives were considered an opportunity to facilitate participants' and their fellow countrymen's empowerment, reflecting acceptance of such measures. Empowerment was rooted in experience of compassionate care based on respectfulness, voluntariness and healthcare access; enabling decisional and health control. Furthermore, preventive measures were perceived as deeds signalling care for them and their health.

I think it's a good deed and thank you many times (Participant 9)

I think it's good because it's humanitarian aid. You get examined and diseases are prevented (Participant 6)

Considering prevention, a ‘humanitarian deed’ indicated confidence in the Danish healthcare system's efforts to safeguard their best interest; perceptions rooted in experiences of, for example, paternalism and denial of healthcare access in their native countries, causing feelings of disempowerment.

My mother was very ill, so I took her to the hospital. But we were refused access to medical care at the hospital's front door (Participant 3)

Thus, they were particularly grateful for having access to a healthcare system based on equality and compassion.

Fortunately, we came to Denmark where we have received care. They are not doctors; they are angels! (Participants 5)

Compassionate care was also promoted through experienced empathy and healthcare professionals accommodating their needs and taking co‐responsibility for their health. Such experience empowered them as they achieved greater health control. Moreover, the display of co‐responsibility enhanced cultural acceptability towards prevention.

Here in Denmark, they respect people. One should participate and get checked, it's for one's future! (Participant 9)

We interpreted participant 9's statements on Danish values as being of a humanitarian nature to reflect a higher educational level and being integrated (Table 1). Voluntariness prompted the ethical acceptability of prevention, which further promoted feelings of empowerment by delegating decisional control. Additionally, prevention was considered worthwhile from a societal perspective.

If I get heart problems and need surgery, it costs more than prevention. I agree with the idea and think preventive studies are really good and you should participate. It's also good for the economy (Participant 4)

The relevance of prevention seems rooted in the Arabic proverb ‘prevention is better than treatment’—a proverb participants repeatedly used. We interpret that finding prevention relevant as expressed in a culturally bounded proverb promoted a feeling of empowerment.

Overall, we argue that the feeling of empowerment was rooted in an ethical and humanistic compassionate care‐based healthcare system facilitating individuals' decisional and health control. Thus, preventive initiatives were perceived as ethically and culturally acceptable. However, empowerment through prevention was challenged by 2 other perceptions as discussed below.

#### 3.1.1 We are both hampered and strengthened by our basic assumptions

Participation was questioned due to overlapping perceptions of the challenges encountered when engaging in prevention. Thus, the participants requested help to make their countrymen acknowledge the relevance of prevention. We interpret that basic assumptions, rooted in their native culture, religion and lived experiences, may hamper and paradoxically also strengthen their abilities to engage in prevention. The assumptions were directed towards fearing authorities and a divine power that arranges life for them; assumptions affecting how they act in life and perceive their health.

The participants explained how immigrants' living conditions promoted assumptions through which they perceived life.

If you are sending a letter like the one you sent to me in Arabic … blah blah blah and terms. Its sounds very serious! Maybe they think that's now they come to do experiments on us. It's this kind of mentality they have (Participant 7)

Fear of being exposed to human experiments reflects traumatic experiences have scarred them for life. Fear towards native authorities also produced unwillingness to share personal information.

They think more about how they can dodge [participation] because they think that you send the information on to the municipality or something like that. It's because of how we have lived in our [bloody] country of origin… in our homeland, we keep personal information secret. You may say it's a hidden culture (Participant 10)

Thus, evasion may be the first reaction to being invited; reflecting an emotional reaction that further influences the relevance and cultural acceptability of any initiative. A distrust so inherent that one participant expressed:

Mistrust is something we have had in our home country, how can we restore trust? Does it need an entire generation that is more integrated in Denmark? (Participant 10)

This quote reflects that distrust is ingrained in their identity. Nevertheless, using such phrases, they demonstrated that re‐establishing trust was possible. It was heartbreaking for them that not all of their countrymen were able to relinquish this fear of and distrust in authorities after coming to Denmark.

It isn't fair that they still live with their line of thinking when living in such a country [Denmark] (Participant 7)

We interpret that this fear gave rise to basic assumptions about health and disease; assumptions that were expressed as ignorance and avoidance of seeking medical care.

If a person has no knowledge about health, it leads to choosing not to participate and make fun of it. This is found especially among my country fellows. Many people get symptoms, but they don't care until they realize that these symptoms are caused by serious illness. People who have not learned about health don't participate and don't see the importance of it (Participant 10)

If a person is unwilling to seek medical care when experiencing symptoms, then prevention may be less relevant. Another trend was an unwillingness to talk about illness. In this context, a cultural metaphor for thinking about illness is ‘causing a headache’. We interpret that “headache” reflects concerns about receiving a positive test result.

My fear is, if anything, hidden, and I will become aware of it. The result! But it's God who decides everything (Participant 8)

Despite believing that God decides everything, including health, the participants advocated for prevention.

God decides everything. All the inside, we know nothing about, only God does. All the inside is something we can't control ourselves, while all the outside is something we can control ourselves (Participant 3)

As such, they may take responsibility to control things on “the outside” that affect their health, which may encourage engagement in prevention. Barring the participant of Somali origin, religion was prominent in affecting how health‐related basic assumptions were expressed. Overall, we found that several assumptions were rooted in fear and distrust; health and disease and unwillingness to think about illness and share information. We interpret that these basic assumptions provide meaning to their life while being predominantly inhibitory from a prevention and health‐promotion perspective as they hamper engagement in prevention.

#### 3.1.2 We need help to achieve coping capabilities enabling us to engage in preventive initiatives

Preventive initiatives were perceived as relevant by representing an individual and societal investment in health. Therefore, participants shared their views on how to make prevention relevant to their fellow countrymen. They identified as a factor in this respect their own and other immigrants' coping capabilities to engage in prevention at all and make an informed decision; capabilities involving critical reflection, linguistic and relational competencies. Knowledge and motivation were found to be central to engaging in prevention. Furthermore, sufficient information promoted a sense of safety.

Participation, for me personally, is promoted by motivation and by being given sufficient information about the test, how to participate and the test procedure … details! It's always good to know what is going to happen (Participant 7)

However, for an invitation to be informative and motivating, it must be understandable and take into account language proficiencies.

Most Arabs over the age of 60 who live in Denmark are uneducated and unengaged; they can't read (Participant 7)

Factors influencing the capacity for critical reflection. Language preferences for invitations varied from Danish to preferring their mother tongue. Favouring their mother tongue was related to recognizing its relevance in avoiding misunderstandings and reducing potential distress. The distress caused by a preventive invitation was suggested remedied through the use of digital decision support.

Those who have studied medicine can make a video where they explain or maybe a website in Arabic from the hospital where they write with us and communicate with us. It will benefit many people (Participant 4)

Participant 4 had undergone heart surgery and used that experience as a basis for his perceptions of preventive measures. A personalised approach, communicating through the immigrants' children, was recommended as an invitation strategy.

I have worked as a communicator and hold experience with people who don't come from Denmark, especially Arabs. Their mentality is that they handle things personally, not in writing, and in a culture‐specific way… therefore direct contact with them is perhaps the only tool to get them to participate in preventive offers. This may happen either through their sons or daughters. The younger ones who also have the Danish culture or speak and understand Danish, they can help passing on the idea to their family. They need to be encouraged to come! (Participant 7)

As such, prevention was communicated through those most trusted. Personalised invitation through collaboration with their GP was also suggested; the participants expressed that next to God, they listened to their GP.

I think it will succeed if it is through the doctor. When they receive something from their general practitioner, they perceive it as if the doctor is keeping an eye on them [caring for them]… they think the general practitioner is responsible for them. Because we always love to follow our leader and the doctor is considered a leader. So, if it's coming through their general practitioner, I think participation is going to flourish more (Participant 7)

This quote expresses trust in the GP. In summary, a person‐centred invitation approach is needed to promote engagement in prevention and informed decision‐making. Given their basic assumptions and coping capabilities, we found it questionable whether Arabic‐speaking immigrants possessed sufficient competencies and resources to engage in prevention. These concerns suggest that their health literacy should be strengthened to ensure inclusive preventive initiatives.

## 4 DISCUSSION

This study aimed to explore Arabic‐speaking male immigrants' perceptions of preventive initiatives to understand how to address inequality in engagement. We found that living in Denmark, these men found preventive initiatives ethically and culturally acceptable. As such initiatives empowered them and their fellow countrymen; enabling them to achieve greater control of their health. The participants found it important to convey that Arab‐speaking men may be a hard‐to‐reach population due to their basic assumptions and coping capabilities. Enabling empowerment must therefore be understood in light of their basic assumptions and coping capabilities, which further influenced their health literacy. The Global Health Promotion defines health literacy as ‘the combination of personal competencies and situational resources needed for people to access, understand, appraise and use information and services to make decisions about health’.^20^

We found prevailing basic assumptions likely to influence health literacy for engagement in prevention. Believing that one's health is in God's hands agrees poorly with a biomedical understanding of disease prevention. Similarly, believing in God was reported as a reason for declining preventive measures amongst Arabic‐speaking immigrants in the study by Abdelmessih et al.^14^ Nevertheless, we found that although believing that God decides health, Arabic‐speaking men may take personal responsibility for their health for which reason prevention was advocated. However, according to our findings, advocating for and engaging in prevention hinges on re‐established trust towards authorities; trust achieved through the experience of compassionate care based on ethical and humanistic dimensions involving healthcare access, respectfulness, voluntariness and catering to one's needs. Similar dimensions were found to be central for compassionate care in healthcare systems in a review by Tehranineshat et al.^21^ Without re‐established trust, Arabic‐speaking men may respond with distrust when receiving a preventive invitation and they may opt out of participation due to fear; a response rooted in traumatic experiences in their native countries. Traumatic experiences are common amongst non‐Western immigrants in Denmark. Hence, Ostergaard et al.^22^ found that 47% reported having experienced torture or traumatic events like war or persecution. We argue that fear‐based assumptions rooted in lived experiences may trigger an emotional rather than cognitive‐based reaction to invitations. As such, fear overshadows the relevance of prevention and hinders participation. In fact, without trust, prevention may neither be acceptable nor relevant.

We found that distrust may also be curbed by involving people with whom a trustful relationship already exists, for example, GPs. Furthermore, Patel et al. found that using health professionals who speak the refugees' language gave the refugees a feeling of speaking to a trusted person with the same cultural and linguistic background.^23^ We argue that trust is central to building culturally appropriate prevention services in which acceptability and thereby accessibility may also be facilitated through intercultural agents. Such agents' key roles are to facilitate trust and underpin empowerment by informing about and interacting with health services.^24^

We found that coping capabilities were likely to influence health literacy for engagement in prevention from an accessibility and acceptability viewpoint. Limited language proficiency hampered information access and thereby also prevented participation. Sending a written invitation was an inadequate approach regardless of the language used because we cannot expect all older immigrants to be literate. Similarly, Abdelmessih et al. found that Arabic‐speaking participants requested verbal information because they were illiterate. Preferring verbal information is also related to the fact that the Arab and Somali communities in Denmark are verbal cultures.^25^ Similarly, we found that Arabs ‘handle things personally, not in writing’. Therefore, an invitation approach involving children and GPs was suggested. Conveying prevention may also be possible through immigrant communities. Accordingly, Tatari et al. found that within Arab and Somali communities in Denmark, people are a central source of information for each other.^25^ Social relationships and networks are a potential resource for health literacy; still, we draw attention to the fact that immigrant laypeople may be unable to convey the relevance of prevention because of their cultural and religious assumptions. Therefore, such an approach may not remedy ethnicity‐based inequality in acceptability and accessibility.

Arabic‐speaking men's response to preventive invitations may be understood within the theory of self‐efficacy. In this theory, an individual's ability to cope is affected by similar previous challenges and emotions related to the specific challenges encountered.^26^ Thus, we argue that prevention is well‐perceived culturally and is ethically acceptable to those with re‐established trust towards authorities. Inversely, those distrusting and fearing authorities respond emotionally on the basis of their basic assumptions. This is inexpedient because when engaging in prevention, the capacity to make an informed rather than an emotional decision is essential. We, therefore, find it urgent to address immigrants' capabilities for decision‐making by strengthening their health literacy. In line with our concerns about Arabic‐speaking men's challenged health literacy, Bo et al. found that 17%–27% of immigrants living in Denmark perceived difficulties in understanding health information.^27^ Even so, a recent Danish survey by Svendsen et al. amongst the general population found inadequate health literacy to be associated with immigrant status only in unadjusted analyses (odds ratio, 1.71; 95% confidence interval, 1.30–2.25).^28^ However, as underscored in a recent review by Urstad et al., the literature on health literacy may not reflect the full picture due to the use of different definitions and instruments.^29^ This problem may also apply to the framework of health literacy. We found education to be a likely determinant of health literacy amongst Arabic‐speaking men. Our findings are supported by the literature; worldwide, 30% of adult Muslim men have no formal schooling.^30^ As such our study illuminates the complexity of promoting equal engagement in prevention.

Although we did not aim to elicit differences and similarities between ethnicities and cultures, we found one distinctive difference. Apart from Somali origin, religion was prominent in how health‐related basic assumptions were expressed. Furthermore, traumatic life experiences were a commonality. These findings are supported by Lechner‐Meichsner and Comtesse amongst refugees from Arabic countries and Sub‐Saharan Africa.^31^ Another commonality was a concern for their fellow countrymen's capabilities to engage in prevention.

In summary, we found that acknowledging basic assumptions and coping capabilities was central to a person‐centred approach catering to diverse populations' needs, preferences and values in accordance with the WHO's definition of acceptability.^3^ We argue that basic assumptions and coping capabilities may influence inclusivity in prevention; a concern that may be addressed by strengthening health literacy.

### 4.1 Implications for addressing ethnicity‐based inequality in the prevention

We found it urgent to address health literacy to promote equal access to prevention and thereby to health. According to the WHO, health literacy is key to informed decision‐making on whether to participate or not, and it empowers individuals and communities alike. Thus, health literacy may be addressed at structural, health professional and individual levels.

To uncover invitees' needs, we recommend investigating their health literacy to design interventions addressing their needs before evaluating any intervention effects. At the structural level, we suggest that prevention initiatives be designed by involving representatives from vulnerable groups. Public involvement is an acknowledged approach to improving the quality and relevance of healthcare alongside improving information readability and facilitating participation.^32^, ^33^, ^34^ At the health professional level, we recommend formal training to facilitate person‐centred care that is sensitive to health literacy, including basic assumptions. At an individual level, health literacy also includes the ability to critically appraise health information; an ability we find likely to be inadequate amongst older immigrants. Digital solutions were suggested to support communication with illiterate people. Such an approach would also support the invitees by giving them access to trustworthy health information. At all levels, we recommend involving trained intercultural agents as the review by Verrept indicated that they may increase participation.^24^ According to our findings, involving laypersons as intercultural agents requires standardised training to pave the way for informed decisions. Finally, an invitation by a GP was found important to enhance inclusivity.

Ethnicity‐based inequality in engagement is a general concern. Our proposed recommendations may be applicable to healthcare services in general. From a practice, research and policy perspective, this study sheds light on Arab‐speaking immigrants' challenges and needs. Our findings support inclusive prevention; responding to ethnicity‐based inequality with the potential to improve health outcomes amongst immigrants at both individual and societal levels.

### 4.2 Methodological considerations

Data were collected during the Covid‐19 pandemic; telephone interviews were used to encourage participation by using technology requiring few technological skills, enabling anonymity and prompting comfortability in disclosing sensitive information^15^, ^35^ However, limitations of telephone interviews are acknowledged. The interviewer was unable to observe nonverbal expressions that support detailed elaboration on perceptions. In telephone interviews, silence and pausing may be experienced as awkward.^36^ Nevertheless, we succeeded in achieving an in‐depth understanding of the research question by using an interpretive analytical approach.

Amongst those approaches, 26% participated, leaving 10 participants in our study. We acknowledge that this is a potential limitation, entailing certain reservations towards our findings. Nevertheless, our study contributed to a valuable understanding of ethnicity‐based disparity in access, acceptance and relevance in prevention. In future research, it would be interesting to explore the perspectives of those who do not participate in preventive measures and of women. In Arabic societies, women may differ from men in terms of health‐related decisions. We found that men may consult their GP. In contrast, women may consult their husband^37^ reflecting a patriarchal structure of society.^38^ The participants in our study expressed an erased patriarchy; one of their motives for participating was safeguarding the female interviewer's career. Similarly, men may position themselves as fathers or enlighteners towards female interviewers.^39^ Both fatherly and enlightener positions influence interactions in interviews; such interview dynamics are yet unexplored.

Although our study population represented distinct countries, life narratives were alike and motives for participating were equivalent to reduce inequality in prevention engagement and help the interviewer. Despite the Arabic dialects varying, no one was excluded from participation. Moreover, the study population represented males in their 60s. These factors enabled us to reach data saturation although the study population was small.

Approaching Arabic‐speaking men, we used a culture‐sensitive and person‐centred approach enabling us to collect empirical data. The interviewer's name appeared from the interview invitation; hence, the candidates were aware that it came from a country fellow. Moreover, the bilingual and bicultural interviewer gave voice to those who were monolingual and ensured cultural appropriateness and sensitivity throughout the research process.

## 5 CONCLUSION

Prevention was perceived as ethically and culturally acceptable alongside personally and socially relevant; and representative of Arab‐speaking men living in Denmark. Acceptability originated from a feeling of strengthened empowerment. Relevance was rooted in viewing prevention as personally and socially beneficial. But Arabic‐speaking men may be a hard‐to‐reach group due to their basic assumptions and capabilities for engaging in prevention; raising concerns related to acceptability and accessibility which are essential to the human right to good health.

Addressing inequality in accessibility, acceptability and relevance in regard to prevention may be promoted through a person‐centred approach embracing invitees' preferences, needs and values. A person‐centred approach will then strengthen Arabic‐speaking men's empowerment; a key element in health‐promoting and disease‐preventive efforts. Inclusivity may further be addressed by strengthening invitees' health literacy through efforts at the structural, health professional and individual levels.

## AUTHOR CONTRIBUTIONS

Study initiators were Marie Dahl, Axel Diederichsen and Jes S. Lindholt. Field data were collected by Rafel Salman Al‐Allaq. Marie Dahl and Rafel Salman Al‐Allaq performed the initial analysis. Marie Dahl and Susanne Friis Søndergaard performed the in‐depth analysis followed by a discussion of the findings in the research group. Marie Dahl drafted the manuscript. All authors contributed constructively during the preparation of the manuscript. All authors have read and approved the final manuscript.

## CONFLICT OF INTEREST STATEMENT

The authors declare no conflict of interest.

## ETHICS STATEMENT

This study was listed in the record of processing activities for research projects in the Central Denmark Region (record no. 1‐16‐02‐351‐20).^40^ Participants received written information in Danish and Arabic; written consent was collected. In Arabic countries, a person's signature is highly sensitive, and signing is perceived as a confidential act. Therefore, only recorded oral consent was obtained amongst two of the participants.

## Supporting information

Supporting information.

Supporting information.

## Data Availability

The data used in the preparation of this article are not available on request. This limitation was implemented to ensure and protect the participants' anonymity and confidentiality.
