# Supplementary material for: Arabic‐speaking male immigrants' perceptions of preventive initiatives: An interview study
Source: Health Expect. 2023 Apr 24;26(4):1618–27. doi: 10.1111/hex.13766 (PMC10349227; doi:10.1111/hex.13766)
Supplement: Supplementary file 2 — Supporting information. [file HEX-26--s001.docx]

**SUPPLEMENTARY MATERIALS**

Supplementary Table S2. Semi-structured interview guide.

S2. Semi-structured interview guide

- the interview guide was informed by existing literature on ethic minorities’ perceptions of preventive initiatives and ethical aspects of the Danish Health Care Act^1^ and screening recommendations.^2^

| Research question | **Theme** | **Questions ^3^** |
| --- | --- | --- |
| Introductory question |  | Would you please start telling me about yourself? |
| What are Arabic-speaking men’s beliefs about the risk and severity of cardiovascular disease (CVD)? | Perceived susceptibility and severity | Who do you think is likely to develop CVD? Do you know anyone with CVD? Sources of knowledge? |
| Which perceptions about health do Arabic-speaking men have? | Health perceptions | Do you worry about your health? If you have questions, where do you search for answers? What do you think? |
| What are Arabic-speaking men’s attitudes towards preventive initiatives in general and for CVD? | Perceived benefits | Have you participated in any screening programmes or health checks with a general practitioner? What do you think of preventive initiatives for CVD and preventive initiatives in general? Have you considered the potential pros and cons of such initiatives? |
| What are Arabic-speaking men’s perceptions of accessibility and acceptability? | Right to health | Do you see any barriers to participation? Do you have any suggestions for eliminating barriers and facilitating participation? How can preventive measures take into account immigrants’ needs and be person-centred? |
| How do Arabic-speaking men experience the Danish healthcare system? | Healthcare-seeking behaviour | How do you feel about the Danish healthcare system versus your native country’s system? Relationship to your general practitioner? When and why do you seek medical assistance? |
| Does invitation for preventive initiatives impact the individual’s integrity, self-determination and respect for the individual? | Ethical aspects related to preventive initiatives | Do you consider participation in preventive initiatives to be voluntary? |

1. The Danish Health Act emphasizes that the healthcare system must ensure respect for the individual person, their integrity and self-determination and ensure, e.g., equal access to healthcare, freedom of choice, easy access to information and that a health service provider does not discriminate against patients due to nationality.
2. The Danish recommendation for any screening initiatives highlights that such initiatives must be publicly acceptable. Furthermore, individual’s autonomy and integrity must be respected.
3. During the interviews explorative, clarifying and interpretive follow-up questions were used to strengthen the trustworthiness of the findings.
